# Supplementary material for: Learning curve and surgical outcome of robotic assisted colorectal surgery with ERAS program
Source: Sci Rep. 2022 Nov 29;12:20566. doi: 10.1038/s41598-022-24665-w (PMC9709162; doi:10.1038/s41598-022-24665-w)
Supplement: Supplementary file 1 — Supplementary Information 1. [file 41598_2022_24665_MOESM1_ESM.zip › LSC Raw data-Ñ[▒K/117/N_ñw╝╨░O▒Kñσ.pdf]

W52 / W52

臺中榮民總醫院
手術室護理記錄

[redacted]

基本資料

- 室別： 開刀房-06室，手術科別： CRS，手術類別： 常規，日期： 2021/02/18
- 進入時間： 0800 ； 麻醉開始時間： 0818 ； Time Out時間： 0933 ； 劃刀時間： 0934 ； 手術結束時間： 1335 送出時間： 1400 ；
- 麻醉方式： GE
  診斷： CA OF RECTUM,L/3,POSTERIOR SP CCRT
  手術名稱： 1.Low anterior resection with colo-anal anastomosis2.Ileostomy3.Laparoscopy4.
  手術局部用藥： NIL
  術前預防性抗生素：
  - 抗生素名稱一： Veterin 1GM IVD由麻醉護理師給予
  - 時間： 0920
  - 上次簽章: 羅媗鈴

  ，追加預防性抗生素：
- 手術醫師： 蔣鋒帆
- 麻醉醫師： VS 王賜洲
-
  - 刷手護士A ： 何佳青，時間： 0800，接替刷手一： 邱儷媃，時間： 1200
  - 流動護士A ： 羅媗鈴，時間： 0800，接替流動一： 廖子晴，時間： 1200

皮膚狀況

A-V Shunt： 無
術前： 完整
  記錄者： 上次簽章: 羅媗鈴
術後： 完整
  記錄者： 廖子晴

手術準備

手術臥式 單一手術臥式

-
  - 手術臥式： 截石臥式
  - 使用附件： 頭圈，腳架
  - 皮膚準備： 10%Aq Betadine，2% Chlorhexidine
  - 電燒： 使用電燒: 電極片位置： 右大腿，記錄者： 上次簽章: 羅媗鈴

止血帶T： 無

植入物

- 植入物名稱/號碼/數量/部位： Ethicon EEA 33mm x 1釘/rectum
  Ethicon Endo GIA 60-4.1mm x3釘/Rectum
  小飛機*1/迴腸造口處

，記錄者： 廖子晴 上次簽章: 羅媗鈴

標本送檢

- 細菌培養 無
- 細胞學 無
- 冰凍切片 無
- 病理標本
  組織名稱一： Rectum
  ，
  組織名稱二： IMA root
  ，
  組織名稱三： Distal cut end
- 截肢 無

標本交付 標本： 1 袋 ， 標本： 0 盒 ， 標本： 2 瓶 ， 細胞學： 0 管 ， Culture Tube ： 0 管 ， 被交付醫師： R 林志安，

- 記錄者： 廖子晴
- 時間： 1215

傷口術後敷料

傷口敷料： 傷口敷料： 無

- 請於圖上標示部位：

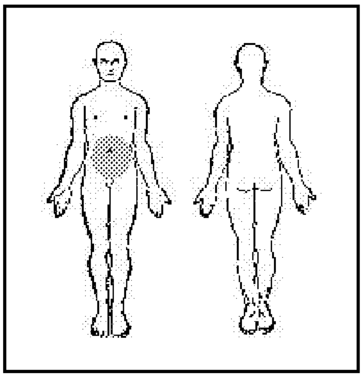

- 傷口敷料： mepore、白紗

導管

導管	天數	導管資訊	插入日期	插入時間	更換日期	拔除日期	拔除時間	拔除原因	預計拔除/更換	預計拔除/更換日期
A-line (1)	1	號碼:20 Ga; 部位:左手;	2021/02/18	0815						
Foley(1)	1	號碼:16 Fr.; 部位:膀胱;	2021/02/18	0850						
Double Lumen CVC(1)	1	號碼:8 Fr.; 深度:15CM; 部位:jugular (右); 象限:RU;	2021/02/18	0905						
Epidural PCA(1)	1	部位:脊髓;	2021/02/18	0820						

用物計數

- 手術中出血量： 150 c.c.
- 手術中尿量： 300 c.c.
-
    - 其它： 無

用物計數： 多切口同時縫合

- 1.器械： 正確 ，2.縫針： 正確 ，3.刀片： 正確 ，4.紗布： 正確 ，5.腹部墊： 未使用 ，6.紗球： 未使用 ，7.PEANUTS ： 未使用 ，8.紗條： 未使用 ，9.絲帶： 正確 ，10.針頭： 未使用 ，11.Vessel loop： 未使用 ，12.Boots： 未使用 ，13.Jelco： 未使用 ，14.棉條： 未使用 ，
    - 第一次：
        - 刷手護士 ： 邱欐媃
        - 流動護士： 廖子晴
        - 時間： 1220

  ,
    - 第二次：
        - 刷手護士 ： 邱欐媃
        - 流動護士： 廖子晴
        - 時間： 1245

  ,
    - 第三次：
        - 刷手護士 ： 邱欐媃
        - 流動護士： 廖子晴
        - 時間： 1250

備註

備註： Olympus NO.1

- 記錄者： 廖子晴 上次簽章: 羅媗鈴
- 時間： 1000

填寫護理人員 廖子晴

正式摘要

		W52 / W52			

doctorRank:B
